# Supplementary material for: Tensile strain-induced softening of iron at high temperature
Source: Sci Rep. 2015 Nov 10;5:16654. doi: 10.1038/srep16654 (PMC4639729; doi:10.1038/srep16654)
Supplement: Supplementary Information [file srep16654-s1.pdf]

Supplementary Information: Tensile strain-induced softening of iron at high temperature

Xiaoqing Li,[1,*] Stephan Schönecker,[1,†] Eszter Simon,[2] Lars Bergqvist,[3] Hualei Zhang,[1,4] László Szunyogh,[2,5] Jijun Zhao,[6,‡] Börje Johansson,[1,7] and Levente Vitos[1,7,8]

[1] *Department of Materials Science and Engineering, KTH Royal Institute of Technology, Stockholm SE-10044, Sweden*
[2] *Department of Theoretical Physics, Budapest University of Technology and Economics, Budafoki út 8., HU-1111 Budapest, Hungary*
[3] *Department of Materials and Nano Physics, KTH Royal Institute of Technology, Electrum 229, Kista, SE-16440, Sweden*
[4] *Center of Microstructure Science, Frontier Institute of Science and Technology, Xi'an Jiaotong University, Xi'an, 710054, China*
[5] *MTA-BME Condensed Matter Research Group, Budafoki út 8., HU-1111 Budapest, Hungary*
[6] *Key Laboratory of Materials Modification by Laser, Ion and Electron Beams (Dalian University of Technology), Ministry of Education, Dalian 116024, China*
[7] *Department of Physics and Astronomy, Division of Materials Theory, Uppsala University, Box 516, Uppsala, SE-75120, Sweden*
[8] *Research Institute for Solid State Physics and Optics, Wigner Research Center for Physics, Budapest HU-1525, P.O. Box 49, Hungary*

Here we give complementary information regarding the details of the calculations, assessments of the employed methodology, and impact of the shape parameter. In addition, we present two figures for the total energy contours for FM and PM states which show the actual deformation path and the related total energy change (numerical values are given in the main document). The material provided here is expected to make it possible to repeat the *ab initio* calculations and Monte-Carlo simulations and give further support for the reliability of the present predictions.

I. COMPUTATIONAL DETAILS FOR T_C AND $m(\tau)$

The change in T_C under uniaxial tension was accounted for by solving the effective Heisenberg Hamiltonian, $\mathcal{H} = -\sum_{i\neq j} J_{ij}\mathbf{e}_i\cdot\mathbf{e}_j$, of classical spins $\mathbf{e}_i$ with exchange integrals J_{ij} employing Monte Carlo (MC) simulations with the UppAsd program [1]. T_C was derived from the crossing points of the fourth order Binder cumulant [2]. Since the magnetisation curves for Fe derived from classical MC simulations do not reproduce the shape of the experimental magnetisation curve at low temperatures due to the use of classical Boltzmann statistics [3, 4], here we use the analytic expression $m(\tau)$ suggested by Kuz'min [5]: $m(\tau) = [1 - s\tau^{3/2} - (1-s)\tau^4]^{1/3}$. The shape parameter s is related to the spin-wave stiffness constant (D) as $s = 0.1758\frac{g\mu_B}{M_0}\left(\frac{k_B T_C}{D}\right)^{\frac{3}{2}}$ [5], where g is the experimental spectroscopic splitting factor [6] and M_0 is the volume saturation magnetisation. For lower than cubic symmetry, D means the geometric average of the principal values of the spin-wave stiffness tensor, $D = (D_x D_y D_z)^{1/3}$ [7]. Here D_α ($\alpha = x, y, z$) were computed from the exchange integrals J_{ij} in a numerically converging way. We applied the regularisation procedure suggested in Ref. [8] to compute D. We evaluated the numerical accuracy of this method to 2 meVÅ^2 related to the choice of the damping parameter. The J_{ij}'s of Fe entering the MC simulations and the computation of D were obtained by means of the magnetic force theorem [9] in the FM state using the EMTO method. In order to check our data, the spin-cluster expansion as implemented in a Screened Korringa-Kohn-Rostoker method [10] and the local spin density approximation was also used to determine the exchange integrals in the PM state. Both routes gave similar results for $\sigma_m(T)$.

TABLE I. Magnetic quantities used to compute the shape parameter s of the bcc phases of Fe and Fe-Co at the theoretical equilibrium volume compared to the available experimental and theoretical data.

	M_0 (kG)	g^a	D (meV Å^2)	T_C^0 (K)	s
Fe	1.85	2.09	244	1066	0.42
			220-287[b]	1015-1270[c]	0.41[d]
	1.77[e]		280-330[f]	1043[g]	0.35(2)[h]
$Fe_{0.9}Co_{0.1}$	1.91	2.08	285	1286	0.43
				1148[i]	

[a] Experiment; Ref. [6].
[b] Refs. [11, 12] and references therein.
[c] Ref. [13] and references therein.
[d] Ref. [4].
[e] Experiment; Ref. [14].
[f] Experiment; Refs. [15–18].
[g] Experiment; Ref. [19, 20].
[h] Fit to experimental magnetisation curve; Ref. [11].
[i] Experiment; Ref. [21].

* xiaoqli@kth.se
† stesch@kth.se
‡ zhaojj@dlut.edu.cn

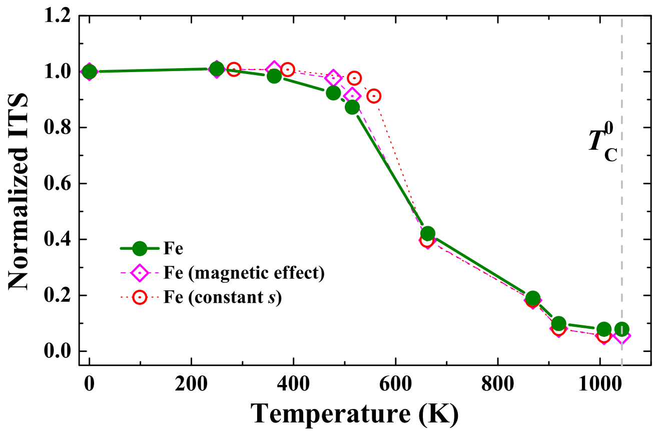

FIG. 1. Comparison between the full and magnetic ITSs (solid circles and open diamonds, respectively) and the ITS obtained by omitting the structure dependence of "s" (open circles). All data are normalised to the ITS of Fe at 0 K (12.6 GPa)

II. ASSESSMENT FOR FE AND FE-CO

To assess the reliability of our computational approach, we first computed the magnetic quantities M_0, T_C, D, and s for bcc Fe. The convergence of all numerical parameters and particularly the k-integration mesh was carefully checked. J_{ij} parameters for all pairs with interatomic distance $\lesssim 8a$ were computed and used for the computation of D. All J_{ij} parameters $\lesssim 5a$ were considered in the MC simulations where the largest simulation box was $30 \times 30 \times 30$ in terms of the conventional unit cell. Our theoretical ground state lattice parameter is $a_{\text{bcc}} = 2.834$ Å, which is slightly below the experimental one (2.867 Å [22]). The present results and the available theoretical and experimental data are summarised in Table SI. The PBE overbinding results in a slightly too large M_0 compared to the experimental value. The computed D and T_C of bcc Fe agree well with the previously published theoretical results, although the theoretical values for D are with the exception of Ref. [12] systematically smaller than the measured ones. The presently derived shape parameter for Fe (0.42) is in line with a previous assessment (0.41, Ref. [4]), but both theoretical assessments are larger than the value fitted to the experimental magnetisation curve (0.35(2) [11]). This discrepancy is mainly due to the underestimated D. For $Fe_{0.9}Co_{0.1}$ alloy, we employed the effective exchange interactions in the random alloy [9], i.e., $J_{ij} = \sum_{\alpha,\beta} c_\alpha c_\beta J_{ij}^{\alpha\beta}$, where $\alpha, \beta =$ (Fe, Co), $J^{\alpha\beta} = J^{\beta\alpha}$, and $c_{\alpha(\beta)}$ is the atomic concentration. Our theoretical data for bcc $Fe_{0.9}Co_{0.1}$ ($a_{\text{bcc}} = 2.848$ Å) listed in Table SI confirms the experimentally determined increase in T_C of dilute bcc Fe-Co alloy.

The PBE error in the equilibrium volume was estimated to affect the ITS by less than 1 GPa at 0 K and 0.2 GPa at 1000 K.

III. IMPACT OF SHAPE PARAMETER s ON $\sigma_{\mathbf{m}}(T)$

In contrast to T_C, we found that the shape parameter s of bct Fe does not significantly determine $\sigma_m(T)$. This is evident from Fig. S1 where the full and magnetic ITSs (solid circles and open diamonds, respectively) are compared to the ITS if the change of s with structural deformation is not considered (open circles). Although the individual quantities T_C and D depend strongly on tetragonality and volume, both follow the same characteristic trend (the one that is displayed for T_C in Fig. 2 in the main document) and their combined effect on s largely cancels out ($s \propto M_0^{-1}(T_C/D)^{3/2}$). We found that M_0 depends rather weakly on both c/a and volume.

IV. IMPACT OF ELECTRONIC SMEARING AND EXPLICIT LATTICE VIBRATIONS

The change in the ITS due to electronic excitations was found to be less than 1 % at $\sim$ 1000 K. The computed explicit phonon contribution decreases the ITS by approximately 5% at high temperature (664 K) and leads to about 1 % change at lower temperature (362 K). Both of the above effects are substantially lower than those due to the magnetic disorder and thermal expansion.

V. TOTAL ENERGY CONTOUR MAPS FOR FM AND PM STATES

Figure S2 displays the total energy of FM and PM states as a function of the lattice parameters a and c, and the corresponding uniaxial deformation paths. For the FM and PM states, the bcc equilibrium is located in a deep and a shallow energy minimum, respectively. The corresponding energy changes of the FM state along the deformation path are much higher than those of the PM state.

[1] B. Skubic, J. Hellsvik, L. Nordström, and O. Eriksson, J Phys:Condens Matter **20**, 315203 (2008).
[2] D. Landau and K. Binder, *Guide to Monte Carlo Simulations in Statistical Physics* (Cambridge University Press, Port Chester, NY, USA, 2000).
[3] N. M. Rosengaard and B. Johansson, Phys Rev B **55**, 14975 (1997).
[4] F. Körmann, A. Dick, B. Grabowski, B. Hallstedt, T. Hickel, and J. Neugebauer, Phys Rev B **78**, 033102 (2008).

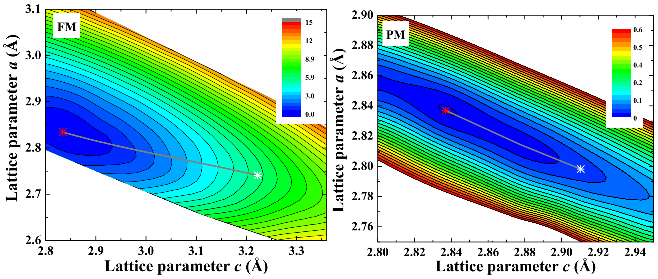

FIG. 2. Total energies (in mRy/atom) for ferromagnetic (FM) and paramagnetic (PM) bct Fe as a function of lattice parameters a and c. The energies are plotted relative to the bcc minimum. The grey solid lines show the uniaxial deformation paths from the unstrained state (bcc, marked by red stars) to the corresponding maximum strain ($\epsilon_{\rm m}$) (marked by white stars). Notice the different energy scales for the two panels.

[5] M. D. Kuz'min, Phys Rev Lett **94**, 107204 (2005).
[6] R. A. Reck and D. L. Fry, Phys Rev **184**, 492 (1969).
[7] C. Herring and C. Kittel, Phys Rev **81**, 869 (1951).
[8] M. Pajda, J. Kudrnovský, I. Turek, V. Drchal, and P. Bruno, Phys Rev B **64**, 174402 (2001).
[9] I. Liechtenstein, M. I. Katsnelson, V. Antropov, and V. Gubanov, J Magn Magn Mater **67**, 65 (1987).
[10] L. Szunyogh, L. Udvardi, J. Jackson, U. Nowak, and R. Chantrell, Phys Rev B **83**, 024401 (2011).
[11] M. D. Kuz'min, M. Richter, and A. N. Yaresko, Phys Rev B **73**, 100401(R) (2006).
[12] A. Szilva, M. Costa, A. Bergman, L. Szunyogh, L. Nordström, and O. Eriksson, Phys Rev Lett **111**, 127204 (2013).
[13] S. V. Halilov, H. Eschrig, A. Y. Perlov, and P. M. Oppeneer, Phys Rev B **58**, 293 (1998).
[14] M. B. Stearns, in *Landolt-Börnstein-Group III Condensed Matter: Numerical Data and Functional Relationships in Science and Technology*, Vol. 19a, edited by H. P. J. Wijn (Springer, Berlin, 1986).
[15] R. Pauthenet, J Appl Phys **53**, 2029 (1982).
[16] R. Pauthenet, J Appl Phys **53**, 8187 (1982).
[17] G. Shirane, V. J. Minkiewicz, and R. Nathans, J Appl Phys **39**, 383 (1968).
[18] C. K. Loong, J. M. Carpenter, J. W. Lynn, R. A. Robinson, and H. A. Mook, J Appl Phys **55**, 1895 (1984).
[19] Z. S. Basinski, W. Hume-Rothery, and A. L. Sutton, Proc R Soc Lond A **229**, 459 (1955).
[20] F. C. Nix and D. MacNair, Phys Rev **60**, 597 (1941).
[21] H. Stuart and N. Ridley, J Phys D **2**, 485 (1969).
[22] P. Villars and L. D. Calvert, *Pearson's handbook of crystallographic data for intermetallic phases*, 2nd ed., Vol. 4 (ASM international, Materials Park, Ohio, 1991).
